# Techno-economic analysis of extraction-based separation systems for acetone, butanol, and ethanol recovery and purification

**DOI:** 10.1186/s40643-017-0142-z

**Published:** 2017-02-13

**Authors:** Víctor Hugo Grisales Díaz, Gerard Olivar Tost

**Affiliations:** 1 School of Chemical Engineering and Advanced Materials, Newcastle University, Newcastle upon Tyne, NE1 7RU UK; 2 Control y Percepción Inteligente, Departamento de Ingeniería Eléctrica, Electrónica y Computación, Universidad Nacional de Colombia, Cra. 27 No. 64-60, Manizales, Colombia

**Keywords:** Extractive fermentation, Dual extraction, High-temperature extraction, Energy evaluation, Biobutanol

## Abstract

**Background:**

Dual extraction, high-temperature extraction, mixture extraction, and oleyl alcohol extraction have been proposed in the literature for acetone, butanol, and ethanol (ABE) production. However, energy and economic evaluation under similar assumptions of extraction-based separation systems are necessary. Hence, the new process proposed in this work, direct steam distillation (DSD), for regeneration of high-boiling extractants was compared with several extraction-based separation systems.

**Methods:**

The evaluation was performed under similar assumptions through simulation in Aspen Plus V7.3^®^ software. Two end distillation systems (number of non-ideal stages between 70 and 80) were studied. Heat integration and vacuum operation of some units were proposed reducing the energy requirements.

**Results:**

Energy requirement of hybrid processes, substrate concentration of 200 g/l, was between 6.4 and 8.3 MJ-fuel/kg-ABE. The minimum energy requirements of extraction-based separation systems, feeding a water concentration in the substrate equivalent to extractant selectivity, and ideal assumptions were between 2.6 and 3.5 MJ-fuel/kg-ABE, respectively. The efficiencies of recovery systems for baseline case and ideal evaluation were 0.53–0.57 and 0.81–0.84, respectively.

**Conclusions:**

The main advantages of DSD were the operation of the regeneration column at atmospheric pressure, the utilization of low-pressure steam, and the low energy requirements of preheating. The in situ recovery processes, DSD, and mixture extraction with conventional regeneration were the approaches with the lowest energy requirements and total annualized costs.

**Electronic supplementary material:**

The online version of this article (doi:10.1186/s40643-017-0142-z) contains supplementary material, which is available to authorized users.

## Background

The interest in biobutanol production by acetone, butanol, and ethanol (ABE) fermentation is increasing because butanol and ABE mixture are considered as an alternative biofuel (Veloo et al. 2010; Kumar et al. 2012). Butanol is the primary inhibitor in ABE fermentation and causes total inhibition at concentrations between 13 and 19 g/l (Xue et al. 2013). In order to reduce butanol inhibition, integrated fermenters have been proposed.

In these processes, butanol is selectively separated from the fermenter (Qureshi and Maddox 2005; Qureshi et al. 2005; Lu et al. 2012; González-Peñas et al. 2014b; Liu et al. 2014; Cabezas et al. 2015). An integrated fermenter allows using a higher substrate concentration. Therefore, the performance of fermenter can be increased and wastewater and energy requirement of downstream and treatment are reduced. Integrated fermenters with liquid–liquid extraction or extractive fermentations are one of the recovery options with lower energy requirements reported in the literature (Groot et al. 1992; Qureshi et al. 2005; Oudshoorn et al. 2009). Solvent selection is involved because several conditions are necessary for the extractant (Kraemer et al. 2011), such as biocompatibility, non-emulsion forming, easy regeneration, high selectivity, low viscosity, high butanol distribution, availability, and low cost. Therefore, several extractive systems have been proposed (Xu and Parten 2011; Kraemer et al. 2011; Grady et al. 2013; Kurkijärvi et al. 2014).

In extractive salting-out, salt solutions or pure neutral, acid, or basic salts (Xie et al. 2013) are proposed as extractants. This is an external process; hence, the productivity of reactor does not improve. The regeneration of the salt is the main disadvantage of the salting-out process. Due to the low concentrations of butanol, in salting-out processes, high energy requirements [21.9 MJ/kg-butanol (Xie et al. 2015) or 28.5 MJ/kg-butanol (Xie et al. 2013)] are required to evaporate the water and unrecovered organic solvents from the salt solution.

Dual extraction (DEx) is proposed using toxic solvents with high butanol distribution coefficient (Kurkijärvi et al. 2014). The toxic extractant is removed with a biocompatible solvent before recirculating the aqueous phase in the fermenter. DEx has been used with a high butanol distribution extractant [Decanol (DAL) (7.1) or octanol (10)] and mesitylene as the biocompatible extractant. DAL was the most promising extractant (Kurkijärvi et al. 2014).

Low-energy fermentation with high-temperature extraction (HTE) (Kraemer et al. 2011) has been proposed for ABE production. An example of high-temperature extraction, using mesitylene as extractant, is the configuration suggested by Kraemer et al. (2011). Mesitylene has a mass partition coefficient of butanol of 0.86 at 30 °C and 3.0 at 80 °C (Kraemer et al. 2011). Therefore, in HTE, when extraction is performed at higher temperatures than fermentation (usually 30–37 °C), less extractant is needed. High selectivity (1970) and medium boiling temperature (180 °C) are the main advantages of mesitylene.

In HTE or DEx systems, the fermenter productivity probably does not increase with respect to continuous process because a high-temperature (80 °C) or toxic extractant would kill the fermenting bacteria. This effect can be avoided with the recirculation or immobilization of biomass. However, the increase in productivity will be achieved through the biomass concentration system. In fact, fermenters with biomass concentration by recirculation or immobilization achieved the highest productivity reported in the literature (Köhler et al. 2015).

Oley alcohol (OAL) is the most studied extractant to carry out in situ extractive fermentation; it has an acceptable butanol distribution coefficient [3.8 (Matsumura et al. 1988)], high selectivity (>300), and it is biocompatible (Evans and Wang 1988). Biocompatibility is the most advantageous characteristic of the extractant because it is used in situ and butanol productivity of fermentation can be increased. However, the high boiling temperature (360 °C) of OAL hinders the extractant regeneration. Therefore, it requires high amounts of preheating, low-pressure distillation, and high-pressure steam.

The combination of toxic solvents and non-toxic OAL has been proposed to decrease the boiling temperature and viscosity of biocompatible extractants, increasing the butanol distribution coefficient (Evans and Wang 1988; Bankar et al. 2012). The mixing ratio is limited by the biocompatibility of toxic extractant. DAL has frequently been proposed in an OAL–DAL mixture of 80–20 wt%. However, non-toxic ratios as large as 60/40 wt% have been reported (Evans and Wang 1988).

Butamax (TM) Advanced Biofuels^®^ developed processes to reduce the boiling point of high-temperature extractants in a regeneration extractant column for isobutanol production (Xu and Parten 2011; Grady et al. 2013). In these patent processes, the aqueous phase from a decanter is recycled to the top of the regeneration column. The supplementation of this aqueous phase allows the recovery of butanol and water from the top and the bottoms, respectively. The high composition of water in the bottoms of distillation column reduces the boiler temperature of the regeneration column. The energy requirement of this regeneration system was between 4.9 and 5.9 MJ/kg-isobutanol. This regeneration system has not been studied for ABE recovery.

An alternative method for regeneration of high-boiling extractants was proposed in this work. The proposed method was called direct steam distillation (DSD). Steam was fed in the bottom of the regeneration column, and water from decanter was not recirculated. DSD can operate at atmospheric pressure using low-pressure steam. Atmospheric pressure operation favors the energy integration because condensation heat can be employed in reboilers of low-pressure columns. Simultaneously, the size of the preheating unit was reduced.

The extractive systems studied in this work were HTE, DEx, conventional extraction, mixture extraction (MEx), and DSD (new process). To our knowledge, MEx using OAL–DAL (80–20 wt%) has been not evaluated economic and energetically in the literature. Due to the different assumptions of the energy requirement reported in the literature (Ezeji et al. 2005; Kraemer et al. 2011; Kurkijärvi et al. 2014; Outram et al. 2016), the selection of the lowest energy system for extractive fermentation is difficult. The main objective of this paper was to select the lowest energy and expensive extractive process for ABE recovery from fermentation. Therefore, in this work, the energy and economic evaluations were performed at similar assumptions.

## Methods

ABE process was simulated in Aspen Plus V7.3^®^. The flash units, distillation, stripping columns, and compressor units were simulated with UNIQUAK-RK. Binary parameters of butanol–water from Aspen Plus V7.3^®^ are not adequate for simulation of vacuum units (Mariano et al. 2011). For this reason, their binary parameters were taken from (Fischer and Gmehling 1994). Decanters were simulated with NRTL. The thermodynamic model of decanters was different because in these units the binary parameters for liquid–liquid equilibrium were used. The missing parameters of NRTL and UNIQUAC (for instance, acetone and OAL binary parameters) were estimated from UNIFAC.

In liquid–liquid extraction column, UNIFAC-LL (Table 1) was used because in Aspen Plus^®^ the binary parameters of NRTL or UNIQUAC for the extractants studied in this paper are not based on experimental data, and UNIFAC-LL has a high accuracy in the prediction of butanol extraction by decanol and oleyl alcohol [Table 1; (Kurkijärvi et al. 2014)]. As UNIFAC is not accurate in the simulation of mesitylene extraction (Kraemer et al. 2011) (Table 1), it was simulated with a constant distribution coefficient of butanol, acetone, and ethanol of 2.2, 0.83, and 0.1 (Kraemer et al. 2011), respectively. CO_2_ and H_2_ were simulated as Henry’s components.

**Table 1:** Solvent properties of extractants studied in this paper

Extractant	ABE	Distribution coefficient		Biocompatible
		Experimental	UNIFAC-LL	
Decanol (Kurkijärvi and Lehtonen 2014)	Ethanol	0.86–0.54 (Offeman et al. 2008)	0.56	No
	Acetone	0.6	1.2	
	Butanol	7.2	7.1	
Mesitylene (Kraemer et al. 2011)	Ethanol	0.1	0.43	Yes
	Acetone	0.83	0.43	
	Butanol	2.2	0.76	
Oleyl alcohol (Matsumura et al. 1988)	Ethanol	0.34	0.40	Yes
	Acetone	0.28	0.74	
	Butanol	3.8	3.9	

The stage number and extraction efficiency (butanol) of all liquid–liquid extraction columns, based on heuristic, were five and 0.8, respectively. Similar stages were proposed evaluating DEx by Kurkijärvi et al. (2014) (four stages of extraction and 100% of efficiency). A specific substrate was not studied because ABE productivity was not calculated. A stoichiometric ABE ratio of industrial production in China was used in this paper (ABE molar basis 2/3/1) (Ni and Sun 2009). Therefore, the stoichiometric reaction is

1 $$11{\text{C}}_{6} {\text{H}}_{12} {\text{O}}_{6} \to 6{\text{C}}_{4} {\text{H}}_{10} {\text{O}} + 4{\text{C}}_{3} {\text{H}}_{6} {\text{O}} + 2{\text{C}}_{2} {\text{H}}_{6} {\text{O}} + 16{\text{H}}_{2} + 26{\text{CO}}_{2} + 4{\text{H}}_{2} {\text{O}}.$$

The fermentation temperature in all cases was 30 °C. The operation of the process is continuous. The concentration in the feed must be limited to possible the presence of solids (e.g., lignin, cellulose, hemicellulose, or ash) and toxic compounds (e.g., furans, organic acids, or phenolic compounds), and the availability of substrate concentration of the real substrate selected (Ezeji et al. 2005; Grisales Díaz and Olivar Tost 2016a). Therefore, a substrate concentration of 200 g/l was selected for the baseline scenario. The conversion and butanol concentration in the fermenter in the baseline scenario were 80% and 10 g/l, respectively.

The comparison of energy requirements of integrated fermenters reported in the literature is difficult because the energy requirements change in reference at assumptions (Outram et al. 2016). For this reason, several concentrations and conversions and one ideal simulation were studied. The ideal evaluation was simulated as proposed by Kraemer et al. 2011: ABE (not glucose) and water were fed to the fermenter without bleed stream (therefore, the water concentration of the substrate is equivalent to water selectivity of the extractant); efficiencies for extraction and distillation columns were of 100%; and nil pressure drop.

The recycle of vinasses was obtained with a ratio of 80 kg-total/kg-ABE. Recovery of solvent from extraction column will be more feasible at lower ratios of broth/OAL. However, the fuel requirement and extractant cost increase. An adequate solvent flow of OAL must be selected through optimization of a pilot-scale system in future work. The Murphree efficiency in the distillation columns was 0.7. Distillation columns were simulated with sieve trays, and pressure drop was calculated with tray rating.

The total annualized cost (TAC) and fuel requirement were calculated with the methodology reported by Grisales Díaz and Olivar Tost (2016a). Extractant loss was included in TAC calculation. Efficiency in steam production with respect to fuel was fixed to 0.9 (Grisales Díaz and Olivar Tost 2016a). CO_2_ production is directly proportional to fuel burn (Jonker et al. 2015). Therefore, fuel savings is proportional to CO_2_ savings. Energy ideal efficiency of separation (IES) of the system was calculated using the following equation Grisales Díaz and Olivar Tost (2016b):

2 $${\text{IES}} = \frac{{R_{\text{S}} \cdot ({\text{LHV}} - H_{\text{S}} )}}{{{\text{LHV}}_{\text{Glucose}} }},$$

where LHV is the lower heating value of solvents and hydrogen (MJ-fuel/kg-solvent), *H*_S_ is the energy consumption of the separation (MJ-fuel/kg-solvent), *R*_s_ is the solvent yield, and LHV_GLUCOSE_ is the lower heating value of glucose, 16.45 MJ/kg (Ruggeri et al. 2015). The energy efficiency was considered ideal because only the energy requirement of recovery and purification systems was calculated. The yield, *R*_s_, was the ABE product (g) per mass (g) of substrate fed. ABE yield is calculated from stoichiometric (Eq. 1) of biocatalyst and conversion.

Installed equipment costs were calculated based on the equations reported by (Douglas 1988). Marshall and Swift equipment cost index (M&S) was 1536.5 (Kim 2015). Equipment was simulated using stainless steel materials. Installation cost of each extraction stage was performed in a pressure vessel with height/diameter ratio of three. Total residence time (aqueous and organic phase) was 0.5 h because experimentally it was found that this is the necessary contact time for an efficient extraction (Bankar et al. 2012). A minimum approach temperature of 10 °C of heat exchangers was performed. Parameters cost used in the economic evaluation are shown in Table 2 (Mussatto et al. 2013; Zauba 2015).

**Table 2:** Parameters used in economic evaluation

Unity	Valor	Unity
Low-pressure steam (3 bar)	2.2	$/tonne (Mussatto et al. 2013)
Mid-pressure steam (30 bar)	7.9	$/tonne (Mussatto et al. 2013)
High-pressure steam (105 bar)	11.8	$/tonne (Mussatto et al. 2013)
Oleyl alcohol	4.3	$/kg (Zauba 2015)
DAL	2.1	$/kg (Zauba 2015)
Mesitylene	2.9	$/kg (Zauba 2015)
Cool water	0.06	$/tonne
Electricity	0.095	$/kWh
Operation time (*t*_o_)	8150	h
Production flow	5000	kg-ABE/h
Time of return investment (*t*_ri_)	5	Year

Stage extraction cost was not calculated for biocompatible extractants because the fermenter productivity with biocompatible extractants increases with respect to conventional fermentation. For instance, in the extractive fermentation of cane bagasse, with OAL–DAL mixture and cell immobilization, the productivity is increased to 2.5 g/l/h, fivefold higher than that for batch process (Bankar et al. 2012). In other studies, the productivity in extractive fermentation using fed-batch operation, without immobilization, a glucose concentration of 300 g/l and oleyl alcohol as extractant, increased 70% with respect to conventional batch process (Roffler et al. 1988). The fermenter productivity with 100 g/l of glucose concentration and oleyl alcohol or the mixture of oleyl alcohol and ethyl benzoate as extractant was increased 60% with respect to batch process (Roffler et al. 1987).

### Extractant selection

2-Ethyl-1-hexanol (2E1H) is proposed as an extractant for ABE production (Liu et al. 2004; van der Merwe et al. 2013). However, 2E1H toxicity is elevated (González-Peñas et al. 2014a). Additionally, the simulations reported in the literature for ABE production with 2E1H assume infinite selectivity in the extraction. This reduced the required distillation units because there are not azeotropes. However, the selectivity of 2E1H [295 (González-Peñas et al. 2014a) and 330 (Kraemer et al. 2011)] is similar to OAL (>300). For these reasons, this extractant was not studied in this paper.

Hexyl acetate is an extractant evaluated for butanol production (Sánchez-Ramírez et al. 2015; Errico et al. 2016). However, experimental data of biocompatibility or distribution coefficients of butanol extraction by hexyl acetate are not reported in the literature. Additionally, this recovery has been reported with a very high energy requirement (45 MJ/kg-ABE, calculated in this work from reboiler requirement of route D (315 kcal/s) and ABE production of 47.9 lb/h) reported by Sánchez-Ramírez et al. (2015). For these reasons, this extractant was not studied in this paper.

Biodiesel or additives of gasoline (biocompatible extractants) have been used to recover butanol from fermentation (Li et al. 2010; Kurkijärvi and Lehtonen 2014). Therefore, if butanol is used as biofuel, a final recovery system is not needed. Extraction system using gasoline additives has been proposed with DEx (Kurkijärvi and Lehtonen 2014). Methyl *tert*-butyl ether (MTBE) and ethyl *tert*-butyl ether (ETBE) were the best extraction solvents. Additional purification units were not required (Kurkijärvi and Lehtonen 2014). However, ABE obtained with gasoline additives was lower than 2.6%. Consequently, ABE will be a minority additive in gasoline and ABE chemical market is not covered. For this reason, these fuels were not used as solvents in this paper. Alternatively, the solvents for extractive fermentation can be produced from ABE fermentation products in reactive distillation (Kurkijärvi et al. 2016). However, reactive distillation has a high energy requirement (Kurkijärvi et al. 2016), 2.2- to 2.6-fold higher than that for dual extraction (DEx). For this reason, reactive distillation was not studied in this work.

In this paper, HTE, DEx, conventional extraction, MEx, and DSD (new process) were studied. In DEx, Kurkijärvi et al. (2014) proposed mesitylene and DAL as the biocompatible solvent and toxic extractant, respectively. In this work, OAL was selected instead of mesitylene because OAL has a higher boiling point than DAL. Then, DAL can be recovered at the top of its regeneration column (EC2), and only two columns (instead of three) were required for this section. Mesitylene was used in the high-temperature extractive process (Kraemer et al. 2011). Experimentally, mesitylene toxicity is unknown. However, in this work, it will be considered biocompatible due to its low solubility, as proposed by Kraemer et al. (2011). Conventional extraction was simulated with OAL. However, in the evaluation of DSD, OAL and OAL/DAL (80–20%) were the extractants used. The main differences of extraction-based separation systems are shown in the supplemental material (Additional file 1: Table S1).

#### Distillation system

**Fig. 1:**
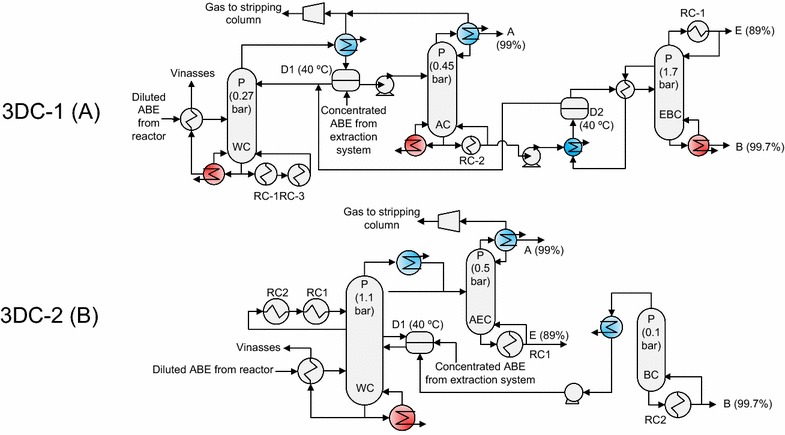
Alternative three distillation columns (3DC) studied in this work. 3DC-1 (**a**) was used in dual extraction, high-temperature extraction, and direct steam distillation. 3DC-2 (**b**) was used in conventional and mixture extraction. D1, decanter. A, B, and E, acetone, butanol, and ethanol, respectively. *P* pressure, *RC* reboiler–condenser

ABE was recovered from vinasses by distillation; it was not by extraction column, due to the low ethanol (Matsumura and Märkl 1984; Offeman et al. 2008) and acetone distribution coefficient of extractants (<1) (Table 1). Two different distillation systems (Fig. 1) were proposed to reduce the fuel requirements of purification of extractive processes. The distillation system used in each fermentation process depended on the condensation temperature of regeneration column, and the condenser or boiler temperature depended on column operation pressure.

The distillation process for HTE, DEx, and DSD had three distillation columns (3DC-1, Fig. 1a). In this system, the heat of condensation of regeneration column of the main extractant was used to apply heat to the reboiler of WC or AC column. For this reason, the columns AC and WC were operated to 0.45 and 0.27 bar, respectively. Vinasses and acetone were recovered in the AC and WC columns, respectively. In the EBC column, butanol and ethanol were recovered at 1.7 bar. In this way, the condensation heat of EBC can be used to provide heat to AC or WC boiler. The stage numbers were selected to avoid an excess of trays. The stage numbers of columns WC, AC, and EBC were 20, 30, and 30, respectively. Organic and ABE dilute phases from the extractive process were fed to the decanter and AC column, respectively.

The design specifications to calculate the boiler ratio of WC and AC columns were the recovery of butanol and (0.999) and acetone (0.99), respectively. The design specifications to calculate the reflux ratio of AC and EBC were the purity of acetone (99 wt%) and ethanol (89 wt%), respectively. While the design specification to calculate the boiler ratio of EBC was the purity of butanol (99.9 wt%), ABE end recovery was 0.97 because ethanol has low relative volatility and acetone recovery from CO_2_ was difficult.

In MEx and conventional, the condensation heat of the regeneration column (EC1) cannot be employed due to its vacuum operation (0.1 bar). For this reason, the final distillation system (3DC-2 system, Fig. 1b) was inverse to 3DC-1. Condensation heat of WC was applied in boilers of columns at vacuum, AEC, and BC. The stage numbers were selected to avoid an excess of trays. The columns trays of WC, AEC, and BC were 40, 20, and 10, respectively (Fig. 1b). The distillation systems studied in this paper have stage numbers lower than that reported in the literature. For example, a conventional five-column distillation system has been evaluated with at total ideal stages of 135 (Mariano et al. 2011; Mariano and Filho 2012) (in this work, the total non-ideal stages of 3DC-2 were 70). The pressure of WC, AEC, and BC columns were 1.3, 0.5, and 0.1 bar, respectively. In the ideal evaluation of MEx (Table 3), the pressures of columns WC and EC1 were 0.3 and 1 atm., respectively. Therefore, in the ideal evaluation of MEx, the condensation energy of EC1 was used in the boilers of AC, WC, and EBC. In a similar way to 3DC-1, the reflux and boiler ratios were fixed to design specifications.

**Table 3:** Energy evaluation at several conditions of extractive processes for ABE recovery from fermentation

Process	Substrate (g/l)	X^a^	ABE yield^b^	Butanol titer (g/l)	ABE titer (g/l)	Pressure EBC (atm.)	*H*_s_^c^	IES	Extractant ratio^d^
DEx	200	0.8	0.311	10.2	27.4	1.7	6.9	0.56	10.1
	200	1	0.388	10.3	30.1	1.7	5.8	0.76	9.8
	200	1	0.388	8.3	23.7	1.7	6.8	0.74	18.7
	300	0.8	0.311	10.2	31.3	1.7	5.3	0.59	10.9
	300	1	0.388	10.1	32.8	1.7	5.5	0.77	10.5
	500	0.8	0.311	10.2	35.3	0.1	4.9	0.60	11.3
	500	1	0.388	10.3	37.0	0.1	4.7	0.79	10.2
	Ideal			10.1	50.0	0.1	2.6 (2.5, [Kurkijärvi et al. 2014)]	0.84	7.8
HTEx	200	0.8	0.311	10.2	23.7	1.7	8.1	0.53	24
	200	1	0.388	10.2	25.3	1.7	7.1	0.73	26
	300	1	0.388	10.2	28	1.7	6.2	0.75	29
	500	1	0.388	10.4	32.7	1.7	6.0	0.76	30
	Ideal			10.3	64.2	0.1	3.6 [3.6, (Kraemer et al. 2011)]	0.81	26.8
MEx OAL/DAL (80–20)	200	0.8	0.311	10	27	0.1	6.6	0.56	12.3
	300	1	0.388	10.2	33.5	0.1	6.2	0.75	14
	500	1	0.388	10.4	39.2	0.1	6.1	0.75	14
	Ideal			10.2	50.2	0.1	2.9	0.83	13.6
DSD OAL	200	0.8	0.311	10.4	26.2	1.7	6.4	0.57	14.4
	200	1	0.388	8.3	23.1	1.7	5.7	0.76	25.0
	300	1	0.388	10.3	29.9	1.7	4.7	0.79	16.8
	500	1	0.388	10.3	34.4	1.7	5.0	0.78	17.8
	Ideal			10.3	49.8	0.1	2.6	0.84	16.0
DSD OAL/DAL (80–20)	200	0.8	0.311	10	27	0.1	6.8	0.56	12.3
	Ideal			10.1	50.2	0.1	3.1	0.82	13.6

*IES* ideal efficiency of separation, *Ideal* substrate concentration as high as extractant selectivity, glucose conversion of 100%, gases in the downstream were not considered, non-pressure drop, trays, efficiency of 100% was assumed

^a^X conversion

^b^ABE yield (g-ABE/g-total-glucose)

^c^ *H*_s_ (MJ-ABE/kg-fuel) energy requirement of the separation system

^d^Solvent ratio, extractant flow/solvent flow

## Results

### High-temperature extraction (HTE)

**Fig. 2:**
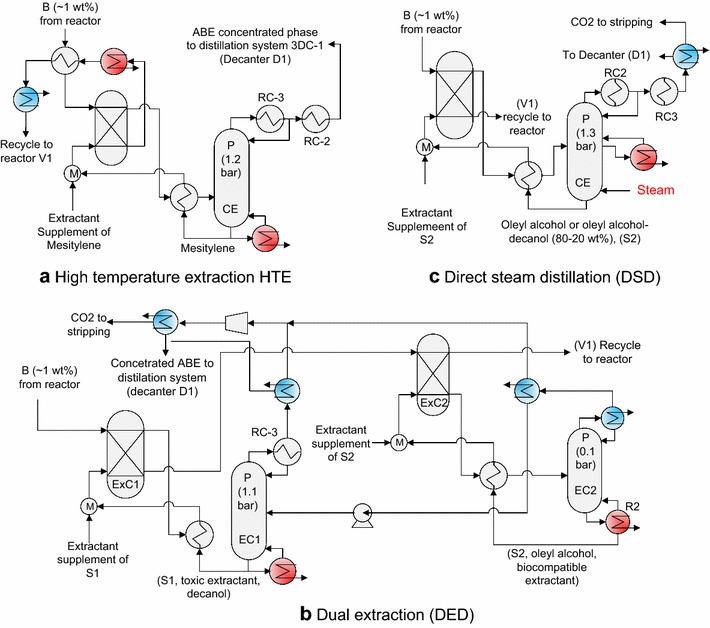
Non-conventional extractive and regeneration configurations. **a** High-temperature extraction. **b** Dual extraction system. **c** Regeneration by direct steam distillation. *B* butanol, *P* pressure, *RC* reboiler–condenser, *D1* decanter

In external extractive systems, there are two options of the bleed stream (Additional file 1: Figure S1). In the first option (a), the bleed stream is direct from the fermenter, while in the option (b) is after the extraction. For this reason, the first part of HTE evaluation was chosen the best purge option. HTE system is shown in Fig. 2a. The extractant in HTE with the option (a) was 33.3% lower than that for the option (b). However, in the option (b), the bleed stream had a less butanol concentration, 2.7 g/l instead of 10 g/l. Therefore, the feasibility of these options depends on the amount of the extractant used and the energy requirement reduction in WC column.

Fuel requirement of WC boiler depended mainly on ethanol concentration of vinasses, not in butanol, due to the low relative volatility of ethanol (~twofold lower than butanol). Ethanol mass fraction using the option (a) and (b) was analogous (5.93 and 5.89 g/l, respectively), due to the low distribution coefficient of ethanol (0.1). Therefore, the energy consumption of WC column using the option (a) and (b) was comparable (4.3 and 4 MJ/kg-ABE, respectively).

Due to the poor butanol distribution of mesitylene (2.2) and the low butanol concentration in the fermenter, the preheating of aqueous and organic streams before extraction was 65% of the total energy. The energy requirements without integration of the options (a) and (b) were 31.3 and 33.5 MJ-fuel/kg-ABE, respectively. The integration heat was favored using an operation pressure for extractant regeneration column EC1 of 1.3 bar because the condensation heat of EC1 was applied in the boilers of vacuum columns AC and WC.

The energy requirement was reduced with energy integration to 8.3 and 8.1 MJ-fuel/kg-ABE for options (a) and (b), respectively. Due to extractant reduction of option (a) [33% lower than option (b)] and similar energy requirement, option (a) was used in all subsequent extractive processes evaluated in this work. The energy requirements of HTE in the baseline scenario were higher than that for ABE recovery from dilute solutions (12.4 g-butanol/l) by heat-integrated distillation (8 MJ-fuel/kg-ABE) (Grisales Díaz and Olivar Tost 2016a).

For comparative purposes, the effects of conversion and substrate concentration in the energy requirements and fermenter design were studied. An increase of conversion in fermenter from 80 to 100% reduced the energy requirement in 11.8%. A less feed and higher recycle, with the increase in conversion, to achieve the fixed broth/ABE ratio used in this work (80 g/g) were required. EBC column was operated at vacuum pressure at substrate concentrations higher than 500 g/l (Table 3). The EBC column was operated at vacuum pressure because the total energy requirement of reboilers of columns WC and AC was lower than that for condenser heat of the extraction column EC1. Consequently, the condensation heat of extraction column was used in the WC, AC, and EBC boilers. In HTE, high substrate concentration required higher solvent ratio and lower fuel consumption to achieve the same butanol concentration in the fermenter (10 g/l) (Table 3).

The ideal assumptions were studied to achieve the minimum energy requirements of HTE. ABE concentration in fermenter under ideal assumptions increased from 23.7 to 62.5 g/l (Table 3). The ABE concentration increased because, under ideal assumptions, bleed stream is not used and the substrate concentration is maximum. The acetone distribution coefficient of mesitylene is 8.3 times higher than that of ethanol (Table 1). For this reason, ethanol concentration in fermenter under ideal assumptions was 4.7 times higher than acetone (9.2 g/l). Acetone and ethanol are much less toxic than butanol (Jones and Woods 1986). In perspective, the addition of acetone at 40 g/l reduces the growth ~50%, and total growth inhibition occurs at a concentration between 50 and 60 g-ethanol/l (Jones and Woods 1986), while the fermentation is inhibited completely at butanol concentrations of approximately 15 g/l. However, this high ABE concentration in fermenter must be toxic for the biocatalyst. Therefore, biocatalysts with a low yield of ethanol and acetone are desired for the operation of HTE at high substrate concentrations.

The minimum energy requirements, under ideal evaluation, of HTE were 5.8 MJ/kg-butanol or 3.6 MJ-fuel/-kg-ABE (Table 3). Heat requirement by mesitylene regeneration was 4.9 MJ/kg-butanol. The energy requirement of the final distillation columns was 1.6 MJ/kg-butanol. However, the condensation heat of extractant regeneration column was used to supply totally the energy requirement of boilers of final ABE purification that operates under vacuum. 2.5 MJ/kg-butanol of condensation was used in the preheating of the HTE system. The reboiler temperature of mesitylene column was 171 °C. Therefore, medium-pressure steam was needed.

### Dual extraction (DEx)

In DEx, two counter-current extraction columns are used. Butanol and the toxic extractant (DAL) were recovered in the columns ExC1 and ExC2, respectively (Fig. 2b). OAL and DAL were fed into the extraction column at ratios of 1.4 and 8.3 kg-extractant/kg-ABE, respectively. Total solvent required for DEx, to achieve a butanol concentration in the fermenter of 10 g/l, was 2.4**-**fold lower than that for HTE process.

The OAL flow needed for 99% recovery of DAL from the broth was only 70 kg/h, a broth/OAL ratio of 5715 g/g. From a practical viewpoint, the extractants at this ratio are difficult of recovery by decantation due to the little organic fraction inside of extraction column (0.019 wt%). In this work, a flow of 7000 kg-OAL/h was chosen arbitrarily. This flow corresponds to 1.7% of total flow fed to extractant column ExC2. The energy requirement of OAL regeneration boiler of base case was only 0.26 MJ-fuel/kg-ABE, 3.8% of total fuel requirement with integration. However, an adequate solvent flow of OAL must be selected through optimization of a pilot-scale system. The volume of each extraction stage of column ExC1 using a residence time of 0.5 h per stage for DEx was 230 m^3^.

Boiler temperatures in OAL and DAL regeneration columns were 272 and 239 °C, respectively. The condensation energy of column EC1, DAL regeneration column, was used to apply heat to WC boiler. Total energy requirements of case base using DEx without integration were 21.4 MJ-fuel/kg-ABE, an energy requirement analogous to process DEx. Total energy requirement with integration was 6.9 MJ/kg-ABE (Table 3). Fuel consumption with heat integration, under similar assumptions, was between 15 and 30% lower than that for HTE.

For comparative purposes, the effect of a reduction in butanol concentration in the fermenter was studied. The extractant flow increased 1.9-fold when butanol concentration in the fermenter was reduced to 8.3 g/l (Table 3). Additionally, energy requirement was increased 14%. The reduction of butanol concentration in fermenter can prevent the strain degeneration and higher operation times can be achieved. For this reason, an optimization of fermenter cost must be performed in future works.

The ideal assumptions were studied to achieve the minimum energy requirements of DED. Energy consumption of ideal evaluation was 2.6 MJ-fuel/kg-ABE using an OAL flow of 70 kg/h. It was an energy consumption similar to that reported by Kurkijärvi et al. (2014) under ideal assumptions (3.8 MJ/kg-butanol or 2.5 MJ-fuel/kg-ABE, calculated in this work assuming 90% efficiency for steam production and A/B/E ratio of 3/6/1). Assumptions proposed by Kurkijärvi et al. (2014) are four ideal stages of extraction, mesitylene and DAL as extractants, A:B:E ratio of 3:6:1, minimum approach temperature of 3 °C, and the same energy requirement of final purification reported by Kraemer et al. (2011), 0.57 MJ/kg-butanol.

### Conventional extraction with extraction mixture (MEx)

MEx achieved an energy requirement without and with heat integration of 21.4 and 6.6 MJ-fuel/kg-ABE, respectively. Energy requirements without and with heat integration were 12.6 and 1.2% lower than pure OAL, respectively. A low energy requirement without heat integration is important to reduce the exchanger area of the process. Energy requirements of MEx were between 6.6 and 6.1 MJ-fuel/kg-ABE at glucose concentrations between 200 and 500 g/l, respectively (Table 3).

Fuel consumption was reduced by 8.1% with an increase of substrate concentration from 200 to 500 g/l. MEx at a substrate concentration higher than 300 g/l was an option with higher energy requirement than that for DEx (Table 3). Given that to condensation heat of EC1 was not used in MEx, this reduction was 3.5- and 3.1-fold lower than that for DEx and HTE, respectively. Energy requirement of extraction mixture was between 1.7 and 23% lower than that for HTE (Table 3). The minimum energy requirements (2.9 MJ-fuel/kg-ABE) of mixture extraction were achieved under ideal evaluation (Table 3).

### Alternative regeneration method with DSD

**Fig. 3:**
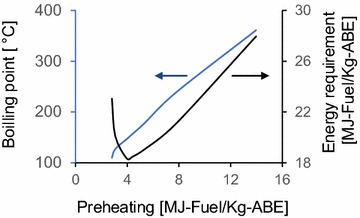
Effect of preheating in energy requirement without integration of regeneration column by direct steam distillation. Top pressure is 1.3 atm.

In the system proposed in this work, steam was fed to bottoms of extractant regeneration column and the boiler was not used (Fig. 2c). In this way, the temperature in the regeneration column decreased. Then, the exchanger area of preheating was reduced. Additionally, the low operation temperature in regeneration column can prevent extractant degradation. Preheating is used to decrease the direct steam flow. However, the maximum temperature in column increased proportionally with respect to preheating (Fig. 3). ABE concentrated from the top of regeneration column was fed to a decanter.

An inflection point takes place at approximately 4.4 MJ-ABE/kg-fuel of preheating, using OAL without energy integration. At this preheated energy, the maximum temperature in the column was around 147 °C, and low-pressure steam (6 atm.) can be used. Without heat integration and direct bleed stream, the total energy requirement for DSD was 18.1 MJ-fuel/-kg-ABE. The energy requirement was reduced to 6.4 MJ-fuel/-kg-ABE through heat integration (Table 3). In mesitylene and DEx process, without heat integration, energy requirements were 1.7- and 1.2-fold higher than DSD with OAL extraction. The energy requirement of DSD was between 3 and 24.2% lower than that for MEx (Table 3).

In contrast to DSD, DEx required high-pressure steam to use the heat of condensation of the regeneration column EC1. In perspective, high-pressure steam is 1.5- and 5.4-fold more expensive than medium and low-pressure steam (Mussatto et al. 2013), respectively. The energy requirements for DSD and OAL of the ideal evaluation were 1.4-fold lower (2.6 MJ-fuel/kg-ABE) than that for HTE (Table 3) and analogous to DEx.

**Fig. 4:**
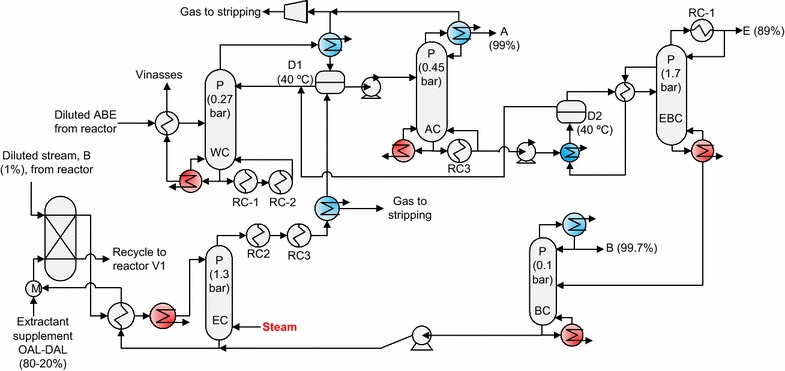
Distillation system for regeneration of DAL–OAL mixture using direct steam distillation. *B* butanol, *A* acetone, *E* ethanol, *P* pressure, *RC* reboiler–condenser, *D* decanter

DSD can be applied with a mixture of OAL–DAL (80–20 wt%) (Fig. 4). Due to low-temperature evaporation of DAL (233 °C) with respect to OAL (357 °C), DAL was partially evaporated. For this reason, the regeneration column was proposed without condenser. Therefore, an additional column was necessary for butanol purification from the extractant–butanol mixture obtained in EBC column (Fig. 4). The minimum energy requirements of DSD without integration were 17.3 MJ-fuel/kg-ABE, 6.3% lower than that for DSD using pure OAL. Extractant was reduced by 14.8% using an OAL–DAL mixture (80–20 wt%) with respect to pure OAL. In the regeneration column, a minimum energy requirement at a pressure of 1.4 bar was obtained with a feed temperature of 168 °C. Energy requirements were 6.6 MJ-fuel/kg-ABE with heat integration, 0.2 MJ-fuel/kg-ABE higher than the energy requirement of DSD with pure OAL.

## Discussion

### Energy evaluation

The energy requirements change drastically with the assumptions of operational conditions and efficiencies of units. Additionally, the energy requirements depend on the selection of final distillation system and heat integration. Hence, a comparison of extraction-based systems with literature data is difficult. In the literature, low energy requirements have been reported with HTE. However, in this work, the lowest energy-efficient system with baseline conditions was HTE (IES equal to 0.53). In this evaluation, the yield of hydrogen from glucose is 0.016 g-hydrogen/g-glucose (stoichiometric ratio of Chinese industrial process, Eq. 1). The hydrogen combustion with this yield was 15.8% of total energy produced. The IES of DSD for the base case was 0.57. The most important factor in IES evaluation was the ABE yield or glucose conversion. For instance, the IES increased from 0.57 to 0.76 when the conversion in DSD and OAL increased from 80 to 100% (ABE yield of).

The IES of ideal evaluation of all extractive systems increased to 0.81–0.84. MEx achieved a similar energy performance to DSD only in the base case. In general, DSD achieved the lowest energy requirement and energy efficiency with and without integration. The high energy integration of DSD was possible thanks to the atmospheric operation of the regeneration column and the low-pressure columns used in ABE purification. In reference to external recovery systems, DEx required less extractant than that for DSD or MEx (Table 3). Therefore, an economic evaluation was necessary.

HTE and DEx are the only extractive processes reported in the literature with lowest energy requirements than that of DSD (6.4 MJ-fuel/kg-ABE). However, these energy requirements are under ideal evaluations. In comparison, Qureshi et al. (2005) reported energy requirements of 7.7 MJ-fuel/kg-ABE [calculated in this work assuming energy efficiency of 0.9 and ABE ratio of *C. beijerinckii* BA101 (ABE of 6/24.6/1)]. Salting-out has been reported with energy requirements between 22 and 25 M/kg-butanol (Xie et al. 2013, 2015), while extraction using hexyl acetate has been reported with energy requirements of 45 MJ/kg/ABE (Sánchez-Ramírez et al. 2015; Errico et al. 2016).

In reference at alternative biofuels, the IES achieved for ABE production by extractive fermentation (100% of conversion) were 3.9 and 5.4% greater than that for ethanol and isobutanol (alternative biofuels) dehydration with double-effect distillation. Double-effect distillation is a heat-integrated distillation system with low energy requirement. In fact, ABE recovery by double distillation has been reported with an energy requirement 20% (8 MJ-fuel/kg-ABE) lower than that for integrated fermenter by pervaporation (Grisales Díaz and Olivar Tost 2016a).

### Economic evaluation

**Fig. 5:**
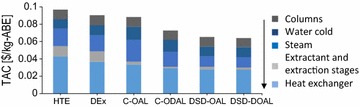
TAC of extractive distillation systems evaluated in this work. C-OAL (conventional using OAL), C-DOAL (conventional using a mixture of OAL and DAL), DSD OAL (DSD using OAL), DSD DOAL (DSD using a mixture of OAL and DAL)

The economic performance of all configurations of extractive fermentation evaluated in this work is shown in Fig. 5. TAC of HTE was 0.097 $/kg-ABE. Total installation cost was 13.4 MM US$. Investment costs of heat exchangers and steam requirement (0.047 and 0.02 $/kg-ABE, respectively) were the most important items in the economic evaluation. Total installation cost of DEx was 10.3 MM US$. TAC and total installation cost of DEx were 4.8 and 23.1% lower than HTE, respectively. The extractant loss and extractant initial investment were the items with less effect in TAC (Fig. 5).

The cost of the extraction column with biocompatible extractant was not calculated, because the productivity with biocompatible extractants increases more than the reduction caused by the volume of stages extraction (0.31 g-ABE/l/h) (Roffler et al. 1987, 1988; Bankar et al. 2012). The volume of extraction can be reduced with the reduction of vinasses recycle or extraction stages. However, it increases the energy requirement (Kurkijärvi et al. 2014). For this reason, optimization of this item must be performed in future work.

In DEx and HTE, the extraction column cost was between 11.5 and 13.4% of TAC. TAC of DSD with pure OAL decreased in 28 and 31.4% with respect to DEx and HTE. The low cost of DSD with respect to DEx was mainly due to the non-cost estimation of extraction column in DSD, the low heat exchanger area, and the low operational costs. Total installation cost of OAL extraction (8.8 MM US$) was reduced to 8.2 MM US$ using an OAL–DAL mixture. TAC of DSD using OAL–DAL mixture was 0.065 $/kg-ABE, 3.5 and 1.4% lower than conventional regeneration using the same mixture and DSD using pure OAL, respectively.

DSD and MEx reduced the TAC in 17.1 and 15.4% with respect to conventional extraction (OAL and conventional regeneration). DSD [with OAL or OAL/DAL (80–20)] or MEx [OAL/DAL (80–20)], in situ recovery units, was more economical than external extraction (DED and HTE). External extraction does not increase the yield or productivity of reactor. Therefore, in this work, an evaluation of fermenter cost was not necessary. The low costs of DSD were due mainly to the utilization of a biocompatible extractant and the low energy requirement without integration or the low exchanger area. An appropriated selection of fermenter conditions of DSD or MEx must be performed through economic optimization. A robust kinetic model for the economic optimization is necessary because low butanol concentrations in reactor required a high extractant flow and energy requirements.

## Conclusions

At a substrate concentration of 200 g/l, HTE and DED were more expensive, and with higher energy requirements than the in situ recovery processes, MEx and DSD. In all evaluated cases, DSD was the process with lower energy requirements and the less expensive. Energy integration of DSD was higher than other extractive processes due to the atmospheric operation of the regeneration column. The less expensive cost of DSD was mainly due to the utilization of a biocompatible extractant and the low energy requirement without integration or the low exchanger area. The ABE yield was the item most important in the energy efficiency calculation of biofuel recovery.

## Additional file

**Additional file 1.** Additional table and figure.

## Abbreviations

AC: column for acetone recovery
DAL: decanol
DEx: dual extraction
DSD: direct steam distillation
EBC: column for ethanol and butanol recovery
EC1: regeneration column of toxic extractant
EC2: regeneration column of biocompatible extractant
ExC1: extractive unit to recover toxic extractant
ExC2: extractive unit to recover ABE
*H*_S_: energy requirement of separation system
HTE: high-temperature extraction
IES: ideal efficiency of separation
LHV: lower heating value
MEx: mixture extraction
OAL: oleyl alcohol
*R*_s_: solvent yield
TAC: total annualized cost
WC: column for ABE recovery from vinasses
2E1H: 2-ethyl-1-hexanol
