# Supplementary material for: Techno-economic analysis of extraction-based separation systems for acetone, butanol, and ethanol recovery and purification
Source: Bioresour Bioprocess. 2017 Feb 13;4(1):12. doi: 10.1186/s40643-017-0142-z (PMC5306422; doi:10.1186/s40643-017-0142-z)
Supplement: Supplementary file 1 — Additional file 1. Additional table and figure. [file 40643_2017_142_MOESM1_ESM.docx]

**Supplemental material**

| **Table S1** Differences in simulation of extraction-based separation systems for acetone, butanol and ethanol recovery and purification | | | | | |
| --- | --- | --- | --- | --- | --- |
| Method | Extractants | Classification | Property method of extraction system | Pressure of regeneration system | End distillation system |
| High temperature extraction | Mesitylene | External  (toxic, high temperature) | Constant distribution coefficient (80^o^C) | 1.2 atm | 3DC-1  (Fig. 1. A) |
| Dual extraction (two extractive columns) | Oleyl alcohol and decanol | External  (toxic, pure decanol is fed to fermenter) | UNIFAC-LL | Oleyl alcohol (0.1 atm); Decanol (1.2 atm) | 3DC-1 (Fig.1 A) |
| Mixture extraction | Oleyl alcohol and decanol mixture (20-80 wt %) | *In situ* (Biocompatible  extractant) | UNIFAC-LL | Vacuum (0.1 atm) | 3DC-2  (Fig. 1 B) |
| Conventional | Oleyl alcohol | *In situ* (Biocompatible  extractant) | UNIFAC-LL | Vacuum (0.1 atm) | 3DC-2  (Fig. 1 B) |
| Direct steam distillation | Oleyl alcohol and Oleyl alcohol and decanol mixture (20-80 wt%) | *In situ* (Biocompatible  extractant) | UNIFAC-LL | Atmospheric (1.2 atm) | 3DC-1  (Fig. 1 A) |

**Fig. S1.** Alternatives of bleed stream in external extractive fermentation
